# Endophilin-A/SH3GL2 calcium switch for synaptic autophagy induction is impaired by a Parkinson’s risk variant

**DOI:** 10.1080/15548627.2023.2200627

**Published:** 2023-04-17

**Authors:** Marianna Decet, Sandra-Fausia Soukup

**Affiliations:** a VIB-KU Leuven Center for Brain & Disease Research, Leuven, Belgium; b Department of Neurosciences, Leuven Brain Institute, Mission Lucidity, KU Leuven, Leuven, Belgium; c Univ. Bordeaux, CNRS, IMN, UMR 5293, Bordeaux, France

**Keywords:** Neuronal activity, calcium, autophagy, Parkinson disease, neurodegeneration, SH3GL2/ Endophilin-A

## Abstract

At the synapse, proteins are reused several times during neuronal activity, causing a decline in protein function over time. Although emerging evidence supports a role of autophagy in synaptic function, the precise molecular mechanisms linking neuronal activity, autophagy and synaptic dysfunction are vastly unknown. We show how extracellular calcium influx in the pre-synaptic terminal constitutes the initial stimulus for autophagosome formation in response to neuronal activity. This mechanism likely acts to rapidly support synaptic homeostasis and protein quality control when intense neuronal activity challenges the synaptic proteome. We identified a residue in the flexible region of EndoA (Endophilin A) that dictates calcium-dependent EndoA mobility from the plasma membrane to the cytosol, where this protein interacts with autophagic membranes to promote autophagosome formation. We discovered that a novel Parkinson’s disease-risk mutation in SH3GL2 (SH3 domain containing GRB2 like 2, endophilin A1) disrupts the calcium sensing of SH3GL2, leading to an immobile protein that cannot respond to calcium influx and therefore disrupting autophagy induction at synapses. Our work shows how neuronal activity is connected with autophagy to maintain synaptic homeostasis and survival.

## Graphical Abstract

**Figure:**
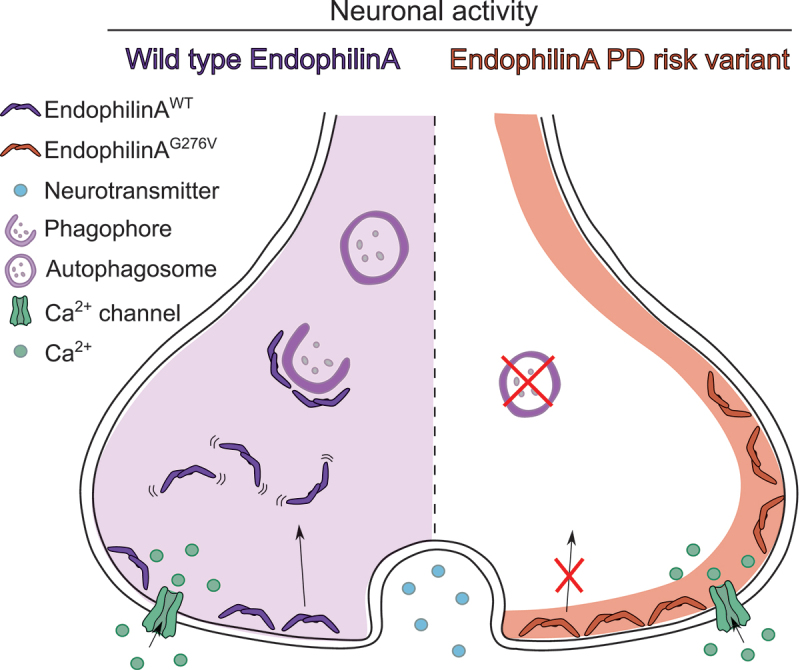

Synaptic terminals are very active and highly dynamic compartments of the neurons, which frequently turn over proteins and membranes to sustain neuronal communication. The unique physiology of synapses makes them susceptible to protein and organelle damage, which could over time result in synaptic demise and ultimately neuronal death. Several studies have shown that synapses locally regulate the turnover of their proteome and organelles as a way to maintain homeostasis independently from the neuronal soma. At synapses, macroautophagy (hereafter called autophagy) is regulated, in addition to the autophagic core machinery, by the synapse-enriched proteins SYNJ1 (synaptojanin 1), BSN (bassoon) and EndoA (Endophilin-A)/SH3GL2 (SH3 domain containing GRB2 like 2, endophilin A1).

Recent studies have shown that autophagosome formation at synapses is intimately linked to neuronal activity, likely acting as a rapid feedback mechanism to support synaptic homeostasis during periods of intense neuronal communication. However, the molecular mechanism linking autophagy, neuronal activity and synaptic demise are vastly unknown. At synaptic terminals, neuronal activity leads to the influx of calcium *via* voltage-gated calcium channels. Electrical nerve stimulation or treatment with the calcium-channel agonist Nefiracetam leads, within thirty minutes, to the formation of Atg8-positive autophagosomes at *Drosophila melanogaster* synapses. Our work further points out that mainly extracellular calcium triggers autophagy induction in response to neuronal activity and that this process relies on EndoA ^[1]^. Previous studies showed that calcium dynamics dictate SH3GL2 interaction with DNM1 (dynamin 1) and voltage-gated calcium channels at the plasma membrane. This calcium-dependent response relies on an unstructured, flexible region between the BAR (Bin/Amphiphysin/Rvs) and SH3 (SRC Homology 3 Domain) domains of EndoA. We introduced a mutation in this region to generate EndoA “calcium-mutants” that mimic EndoA interaction under low and high calcium concentrations. We observed that EndoA mutant mimicking the low calcium presence blocked the formation of autophagosomes even upon calcium influx. Conversely, EndoA mutant mimicking the high calcium presence induced autophagosome formation constitutively.

Our biophysical analysis of recombinant wild-type and EndoA protein harboring the “calcium-mutations” showed that these mutations affect the intrinsic structural dynamics of EndoA. Specifically, the mutant protein mimicking the absence of calcium is more rigid, while the mutation mimicking the presence of calcium is more flexible. We postulated that the effect of calcium on EndoA structure is indirect, as EndoA does not directly bind calcium. EndoA is structurally a very flexible protein and changes in its dynamics likely affect its function. We showed that EndoA mutant mimicking the high calcium condition co-immunoprecipitated with less DNM1 than EndoA mutant mimicking the low calcium condition. In line with these results we found that under low calcium conditions EndoA was localized preferentially to the plasma membrane while under high calcium concentration EndoA was more diffused to the cytosol. EndoA calcium dependent change in localization was mimicked by the “calcium mutations”. By performing super-resolution single-particle tracking (sptPALM) imaging *in vivo* in *Drosophila* synapses, we observed that under low calcium conditions EndoA tends to cluster in nanodomains at the plasma membrane and display a slow, confined mobility similar to EndoA mutants mimicking low calcium conditions. Upon calcium influx, EndoA shows increased displacement accompanied by a reduction in nanodomain size, similar to EndoA mutants mimicking high calcium conditions. Therefore, we propose a model in which in basal condition, EndoA is structurally more rigid, less mobile and preferentially localized in clusters at the plasma membrane where it likely interacts with DNM1. During neuronal activity, in response to calcium influx, EndoA gains structural flexibility, becomes more mobile and diffuses to the lumen of the synapse where it drives autophagosome formation (Figure 1, left side).

**Figure 1.**
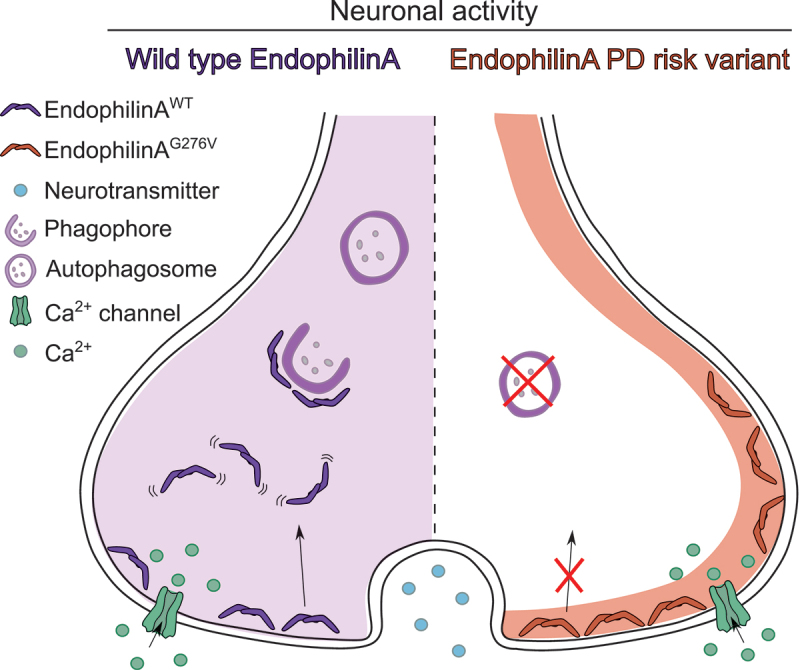
Hypothetical representation of EndoA function in autophagy over the course of neuronal activity. During neuronal activity, calcium influx into the synaptic terminal leads to increased EndoA structural flexibility that in turn leads to the diffusion of EndoA from the plasma membrane to the lumen of the synapse, where the protein promotes autophagosome formation. The Parkinson’s disease risk variant in SH3GL2 restricts its localization to the plasma membrane and consequently blocks autophagy induction in response to neuronal activity.

We and others previously reported that alterations in autophagy, particularly in synaptic autophagy, are associated with neurodegeneration. Consequently, we found that EndoA “calcium-mutants” affects neuronal integrity and *Drosophila* survival. Growing evidence correlates synaptic dysfunction and autophagy to neurodegenerative disorders like Parkinson’s disease. Interestingly, recent GWAS (genome-wide association study) studies have identified variations in the *SH3GL2* locus, coding for SH3GL2 (SH3 domain containing GRB2 like 2, endophilin A1), which are associated with increased risk to develop Parkinson’s disease. One of these variants results in a missense mutation in the disordered, flexible region of SH3GL2. *In vivo* analysis in *Drosophila* showed that the Parkinson’s disease risk variant affects SH3GL2 mobility, subsynaptic localization and calcium influx-dependent autophagy. Similarly, human dopaminergic neurons differentiated from induced pluripotent stem cells (iPSC) harboring the Parkinson’s disease risk variant displayed significantly less autophagosomes in neurites, indicating the effect is evolutionary conserved from flies to humans (Figure 1., right side).

Our work brought us one step forward in the understanding of the mechanism linking neuronal activity, synaptic autophagy and synaptic decay. The results of our work suggest that coupling neuronal activity with synaptic autophagy might act as a feedback loop to regulate turnover rate of proteins and organelles and maintain synaptic homeostasis and function, and to prevent neurodegeneration. Autophagic alterations are common in many neurodegenerative diseases and synaptic decay is thought to precede neuronal loss. Several Parkinson’s disease causative mutations have been found in proteins functioning in synaptic vesicle endocytosis (e.g., SYNJ1, DNAJC6 (DnaJ heat shock protein family (Hsp40) member C6), SNCA (synuclein alpha) and LRRK2 (leucine rich repeat kinase 2)). We previously showed that Parkinson’s disease-causative mutations in LRRK2 and SYNJ1 alter synaptic autophagy. Now we showed for the first time that a Parkinson’s disease risk mutation in SH3GL2 affects autophagy induction at synapses, providing further insight on how synaptic dysfunction is connected to neurodegeneration. This is particularly interesting in light of the fact that SH3GL2 is physically and functionally connected to other Parkinson’s linked proteins participating in autophagy or mitophagy: LRRK2, PRKN (parkin RBR E3 ubiquitin protein ligase) and SYNJ1. A comprehensive characterization of the molecular pathways and intermediates functioning in autophagy at synapses emerges as an essential task to develop promising therapeutic opportunities to treat neurodegenerative diseases.

## List of abbreviations

BAR: Bin/Amphiphysin/Rvs
BSN: bassoon
DNAJC6: DnaJ heat shock protein family (Hsp40) member C6
DNM1: dynamin 1
EndoA: Endophilin A
GWAS: genome-wide association study
iPSC: induced pluripotent stem cell
LRRK2: leucine rich repeat kinase 2
PRKN: parkin RBR E3 ubiquitin protein ligase
SH3: SRC Homology 3
SH3GL2: SH3 domain containing GRB2 like 2, endophilin A1
SNCA: synuclein alpha
sptPALM: single-particle tracking photo-activation localization microscopy
SYNJ1: synaptojanin 1
